# Individual and mixture associations of perfluoroalkyl substances on liver function biomarkers in the Canadian Health Measures Survey

**DOI:** 10.1186/s12940-022-00892-6

**Published:** 2022-09-14

**Authors:** Michael M. Borghese, Chun Lei Liang, James Owen, Mandy Fisher

**Affiliations:** Environmental Health Science and Research Bureau, Health Canada, 251 Sir Frederick Banting, Ottawa, ON K1Y 0M1 Canada

**Keywords:** Hepatotoxicity, Epidemiology, Perfluorinated compounds, Biomonitoring, Liver enzymes, Environmental chemicals

## Abstract

**Background:**

Perfluoroalkyl substances can disrupt hepatic metabolism and may be associated with liver function biomarkers. We examined individual and mixture associations of PFAS on liver function biomarkers in a representative sample of Canadian adults. We explored the potential for effect modification by sex and body mass index, as well as by physical activity level which may attenuate the deleterious effect of PFAS on metabolic disorders.

**Methods:**

We analyzed data from participants aged 20–74 from the Canadian Health Measures Survey. We used linear regression to examine associations between plasma concentrations of PFOA, PFOS, PFHxS, PFNA, PFDA, and PFUDA on serum concentrations of aspartate aminotransferase (AST), gamma-glutamyltransferase (GGT), alkaline phosphatase (ALP), alanine aminotransferase (ALT) and total bilirubin. We used quantile g-computation to estimate associations with a PFAS mixture for each simultaneous, one-quartile change in PFAS concentrations.

**Results:**

Each doubling of PFOA, PFOS, PFHxS, or PFNA concentrations was associated with higher AST, GGT, and ALP concentrations. Each doubling of PFOA concentrations was associated with 16.5% (95%CI: 10.4, 23.0) higher GGT concentrations among adults not meeting Canada’s physical activity guidelines vs. 6.6% (95%CI: -1.6, 15.5) among those meeting these guidelines. Sex and BMI also modified some associations, though to a lesser extent. We did not observe associations between ALT and PFOA (1.2% change; 95%CI: -2.5, 4.9), PFOS (2.2% change; 95%CI: -0.8, 5.3), or PFHxS (1.5% change; 95%CI: -0.4, 3.4). We also did not observe consistent associations for PFDA and PFUDA or with total bilirubin. In quantile g-computation models, each simultaneous one-quartile increase in the PFAS mixture was positively associated with AST (7.5% higher; 95%CI: 4.0, 10.4), GGT (9.7% higher; 95%CI: 1.7, 17.0), and ALP (2.8% higher; 95%CI: 0.5, 5.4).

**Conclusion:**

Higher plasma concentrations of PFOA, PFOS, PFHxS, and PFNA – both individually and as a mixture – were associated with higher serum concentrations of liver function biomarkers. These results contribute to emerging evidence suggesting that higher levels of physical activity appear to be protective against the hepatotoxic effects of PFOA. This work contributes to a growing body of evidence supporting the hepatotoxic effects of PFAS.

**Supplementary Information:**

The online version contains supplementary material available at 10.1186/s12940-022-00892-6.

## Introduction

Perfluoroalkyl substances (PFAS) are a class of highly fluorinated chemicals with lipophobic and hydrophobic properties that are useful for manufacturing a wide range of consumer products, including non-stick cookware, food packaging, personal care and beauty products, fire retardant foams, and carpet treatment applications [1, 2]. Ingestion of contaminated drinking water and food are the primary sources of exposure among the general population [3]. These substances persist in the environment and have been detected in human populations worldwide [1, 4–8]. They bioaccumulate with half-lives ranging from days to years [9] and exposure is nearly ubiquitous among humans [10].

Experimental and mechanistic evidence indicate that PFAS can disrupt hepatic metabolism and promote lipid accumulation in the liver [11, 12]. These effects are at least partially mediated by activation of peroxisome proliferator-activated receptor alpha (PPARα) [13, 14], a regulator of hepatic lipid metabolism. Several other PPARα-independent mechanisms have also been proposed [11, 13], which may be more relevant to PFAS-induced hepatic toxicity in humans. Serum measures of liver function biomarkers are useful clinical indicators of overall liver injury and are commonly used to diagnose non-alcoholic fatty liver disease [15, 16]. These include aspartate aminotransferase (AST), gamma-glutamyltransferase (GGT), alkaline phosphatase (ALP), alanine aminotransferase (ALT) and total bilirubin. In addition, these liver function biomarkers are predictive of several long-term health outcomes, including incident vascular events [17] and diabetes [18], risk of developing hypertension [19] and metabolic syndrome [20], as well as all-cause mortality [21]. Higher circulating serum levels of these liver function biomarkers may represent markers of effect for PFAS hepatotoxicity.

Epidemiological studies have demonstrated deleterious associations between PFAS and liver function biomarkers in both highly exposed [22–24] and general populations [25–29]. These studies have mostly focused on perfluorooctanoic acid (PFOA) and perfluorooctane sulfonate (PFOS). Only one study has considered the cumulative effect of exposure to a mixture of PFAS [12], showing that exposure to a mixture of PFAS was associated with increased risk of liver injury in childhood, including higher concentrations of ALT, AST, and GGT [30]. Further studies on PFAS mixtures and liver enzymes are needed [12]. In addition, associations may be modified by obesity [22, 25, 27] and there is some evidence for sex-specific associations in adolescents [26], although evidence is limited in adults [23, 29]. Physical activity has been shown to attenuate the deleterious effect of PFAS on obesity [31], incident diabetes [32], as well as hypercholesterolemia and hypertriglyceridemia [33]. To our knowledge, the potential for effect modification by physical activity level has not been examined for the association between PFAS and several other liver function biomarkers, including transaminases. Further studies are needed to address these limitations.

Our objectives were to: 1) estimate associations between PFAS and liver function biomarkers in a representative sample of Canadian adults, 2) investigate the potential for effect modification by sex, body mass index (BMI), and physical activity level, and 3) estimate the combined (mixture) association with exposure to PFAS to account for potential confounding by co-exposure to multiple PFAS.

## Methods

### Study design and population

We used data from cycles 1 (2007–2009), 2 (2009–2011), and 5 (2016–2017) of the Canadian Health Measures Survey (CHMS), a nationally representative cross-sectional survey that collects information regarding Canadians’ health through household interviews, questionnaires, and direct measurements. Sampling methodology is described in detail elsewhere [34, 35]. Briefly, a stratified multistage sampling strategy was used to select participants aged 3 to 79 years (6 to 79 years in cycle 1) from 15, 18, and 16 sites for cycles 1, 2, and 5, respectively. The CHMS is representative of 96% of the Canadian population in the ten provinces and excludes those living on-reserves and other Indigenous settlements, institutionalized populations, and full-time members of the Canadian forces. We restricted our analysis to individuals 20–74 years of age who were not pregnant. Participants completed a household interview and were subsequently invited to visit a mobile examination centre where physical health measurements and blood samples were collected. Six PFAS and five liver function biomarkers were analyzed across all three cycles included in this analysis, with some inconsistences – perfluorononanoic acid (PFNA), perfluorodecanoic acid (PFDA), and perfluoroundecanoic acid (PFUDA) were not measured in cycle 1, and ALT and total bilirubin were not measured in cycle 5. The different sample sizes in this paper reflect this inconsistency in data availability. The CHMS was approved by the Health Canada and Public Health Agency of Canada Research Ethics Board [36]. Written informed consent was obtained from all participants included in this analysis [37].

### Measurement of plasma PFAS

A detailed description of plasma PFAS measurement is available for each cycle of the CHMS [1, 38, 39]. Plasma samples were collected using sterile EDTA vacutainer tubes, and were processed, aliquoted, and frozen at the mobile examination clinic. Analysis of PFAS was performed by the Laboratoire de Toxicologie, Institut National de Santé Publique du Québec (Quebec City, Quebec, Canada), which is accredited by the Standards Council of Canada. Analysis was done using ultra performance liquid chromatography coupled to a Waters Xevo TQ-S tandem mass spectrometer (ACQUITY UPLC System; Waters Corporation, Milford, Massachusetts). In the current paper, we included PFAS with > 40% detection, including PFOA, PFOS, perfluorohexane sulfonate (PFHxS), PFNA, PFDA, and PFUDA. Limits of detection (LOD) across the three cycles are provided in Additional File 1 (see Supplemental Table 1).

**Table 1:** Description of plasma concentrations of perfluoroalkyl substances stratified by sex

	Males		Females	
	n^a^	GM (SE)	n^a^	GM (SE)
PFOA (µg/L)	2288	2.2 (0.06)	2365	1.7 (0.05)
PFOS (µg/L)	2288	7.2 (0.26)	2366	4.6 (0.16)
PFHxS (µg/L)	2287	2.2 (0.09)	2365	1.1 (0.05)
PFNA (µg/L)	973	0.67 (0.03)	929	0.64 (0.03)
PFDA (µg/L)	948	0.20 (0.01)	928	0.19 (0.01)
PFUDA	n^a^	%^b^	n^a^	%^b^
< LOD	454	53	414	45
LOD – 75^th^ percentile	306	24	319	32
> 75^th^ percentile	230	23	225	23

*GM* Geometric mean, *SE* Standard error, *LOD* Limit of detection

^a^Sample sizes differ as a result of combining different cycles of the Canadian Health Measure Survey based on data availability

^b^percent within column, calculated using sample weights

### Measurement of liver function biomarkers

Blood samples were centrifuged in the mobile examination clinic laboratory and either refrigerated or kept on dry ice within two hours of collection. Analyses were performed using a Vitros 5600 FS analyzer (Ortho Clinical Diagnostics) [40]. AST, ALT, GGT, and ALP were analyzed using enzymatic multiple point rate/reflectance spectrophotometry and total bilirubin was analyzed using colorimetry/reflectance spectrophotometry. Method coefficients of variation were < 5%.

### Covariates

We identified covariates *a priori* based on previous assessments of risk factors for elevated liver function biomarkers [15], previously identified predictors or correlates of PFAS concentrations [11], as well as previous reports of associations between select PFAS and liver function biomarkers [22–27]. Covariates included: age (grouped by ages 20–29, 30–39, 40–49, 50–59, 60–74 years), race (white and other), biological sex (male and female), BMI (under/normal weight, overweight, and obese with cut-offs at 25 and 30 kg/m^2^, respectively [41]), reported alcohol consumption (regular, occasional, former, and never), smoking (current, former, and never), education (less than secondary, secondary, and at least some post-secondary), accelerometer-measured average moderate-to-vigorous physical activity (i.e., movement intensity at or greater than three metabolic equivalents of task) in minutes/day [42, 43], and household income (low, lower middle, upper middle, and high). The questionnaire item for household income was changed in cycle 5 and replaced by an ordinal unit-less scale that classifies respondents into 10 categories, from 1 (lowest income) to 10 (highest income) [37]. For cycles 1 and 2, we categorized less than C$40,000 as low, C$40,000 to C$59,999 as lower middle, C$60,000 to C$99,999 as upper middle, and C$100,000 or more as high. In cycle 5, we categorized groups 1 to 3 as low, groups 4 and 5 as lower middle, groups 6 and 7 as upper middle, and groups 8–10 as high. We used BMI as a measure of adiposity to allow for direct comparison with previous studies [25, 27, 29], but recognize that BMI may not be the best measure of adiposity and that other measures, such as waist circumference, are more strongly associated with cardiometabolic disease [44].

### Statistical analysis

All analyses were conducted using SAS EG 7.1 (SAS Institute), Standalone SUDAAN 11 (Research Triangle Institute), and R version 4.0.3 (R Core Development Team). The combined cycle 1 and 2 weights file was used for cycles 1 and 2 [45]. Since there is no combined weights file for cycles 1, 2 and 5 and cycles 2 and 5, the individual cycle weight files were used and weights and bootstrap weights were divided by number of cycles combined as recommended by Statistics Canada [45]. A 5% significance level (α = 0.05) was used in all analyses. Participant characteristics, plasma PFAS concentrations, and serum concentrations of liver function biomarkers are presented for men and women, separately, using frequencies or geometric means (SE), as appropriate. PFOA, PFOS, PFHxS, and PFNA were detected in > 98% of samples across cycles, while PFDA was detected in 86% of samples. Values below the limit of detection were assigned a value of one-half of the cycle-specific LOD. The detection frequency for PFUDA was 45%. PFUDA was therefore categorized into three groups: ≤ LOD, LOD–75^th^ percentile, and ≥ 75^th^ percentile and treated as a categorical variable.

In linear regression models, liver function biomarkers were natural log-transformed and PFAS were log_2_ transformed (with the exception of PFUDA) to normalize the distributions and simplify the interpretation of results. Parameter estimates were back-transformed and can be interpreted as the percent change in the outcome for each two-fold increase in the exposure. Residual analysis was implemented to verify statistical assumptions. We explored the potential for non-linearity using 3-knot restricted cubic splines [46]; non-linear associations were not identified and results from the more parsimonious linear models are presented. We tested for effect modification by sex, BMI, and physical activity level by including multiplicative (product) terms in separate models; if interaction terms were not statistically significant (i.e., if effect modification was not observed) the product term was removed from the model. Physical activity level was dichotomized as meeting vs. not meeting the Canadian physical activity guidelines, which recommend that adults obtain ≥ 150 min of moderate-to-vigorous physical activity per week [47]. In addition to the main analysis, we conducted a sensitivity analysis where we excluded individuals who reported being diagnosed with liver disease (*n* = 150). We did not consider this variable as a confounder (either through adjustment or restriction) in the main analysis, since it may be along the causal pathway.

Associations between PFOA, PFOS, and PFHxS and AST, ALP, and GGT were examined using combined data from CHMS cycles 1, 2 and 5, resulting in a sample of size *n* = 4657. The associations between PFOA, PFOS, and PFHxS and ALT and total bilirubin were examined using combined data from CHMS cycles 1 and 2, resulting in a sample of size *n* = 3659. Finally, the associations between PFNA, PFDA, and PFUDA and AST, GGT, and ALP were examined using combined data from CHMS cycles 2 and 5, resulting in a sample of size *n* = 1957. Sample sizes for specific PFAS-liver function biomarker analyses are provided in Additional File 1 (see Supplemental Table 2). Satterthwaite adjusted F test was used in the linear regression models.

**Table 2:** Geometric mean (SE) concentrations of liver function biomarkers and reference ranges by sex

	Males			Females		
	n^a^	GM (SE)	Laboratory Reference range	n^a^	GM (SE)	Laboratory Reference range
AST (U/L)	2281	27 (0.32)	18 – 54	2354	23 (0.21)	18 – 39
ALT (U/L)	1661	35 (0.76)	18 – 78	1721	28 (0.58)	16 – 44
GGT (U/L)	2281	27 (0.57)	12 – 109	2356	20 (0.57)	10 – 54
ALP (U/L)	2270	73 (0.86)	50 – 167	2346	71 (0.81)	44 – 122
Total bilirubin (µmol/L)	1757	7.1 (0.39)	2 – 21	1844	4.3 (0.22)	2 – 17

*GM* Geometric mean, *SE* Standard error

^a^Sample sizes differ as a result of combining different cycles of the Canadian Health Measure Survey based on data availability

We used quantile g-computation, a generalized linear model based implementation of g-computation [48], to estimate the combined (joint) association with exposure to a mixture of PFOA, PFOS, PFHxS, PFNA, and PFDA. This method estimates the parameters of a marginal structural model that characterizes the change in the expected potential outcome for a simultaneous, one quartile increase in all of the exposures in the specified mixture. We did not include PFUDA in the mixture because of the low detection level.

## Results

Sociodemographic characteristics of participants in cycles 1, 2, and 5 of the CHMS included in this analysis are provided in Additional File 1 (see Supplemental Table 3). Plasma concentrations of PFAS, stratified by sex, are presented in Table 1. Concentrations of liver function biomarkers, stratified by sex, are provided in Table 2 along with sex-specific laboratory/statistical reference ranges [40] which reflect the expected range of values from 95% of the broader population [49]. It is important to note that these are not health based reference ranges. For instance, a healthy ALT concentration from individuals without clinical risk factors for liver disease ranges from 29 to 33 U/L for males and 19 to 25 U/L for females [49]. Correlations between PFAS ranged from 0.20 to 0.71 (Additional File 1, see Supplemental Table 4).

**Fig. 1:**
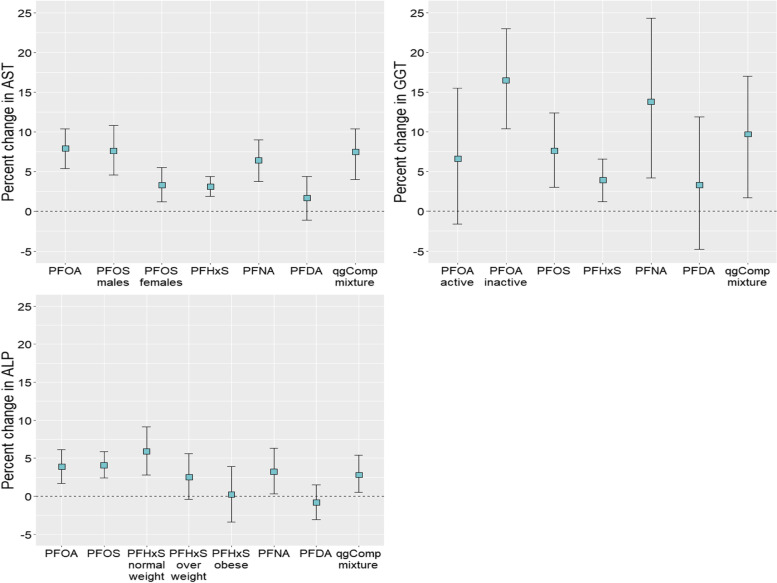
Percent change (95%CI) in AST, GGT, and ALP for PFAS individually and as a mixture. Coefficients for individual PFAS represent the percent change for each two-fold increase in PFAS concentrations. Coefficients for qgComp mixture represents a simultaneous one-quartile increase in PFAS concentrations derived using quantile g-computation. Active and inactive refers to effects among individuals who either meet, or do not meet, Canada’s physical activity guidelines, respectively

In linear regression models, PFOA, PFOS, PFHxS, and PFNA were consistently positively associated with AST, GGT, and ALP (Fig. 1 and Additional File 1, see Supplemental Table 5)). Each two-fold increase in plasma PFOA concentrations was associated with 7.9% (95%CI: 5.4, 10.4) higher AST and 3.9% (95%CI: 1.7, 6.1) higher ALP. Some associations with each of PFOA, PFOS, and PFHxS were modified by sex, BMI, or physical activity levels for different outcomes.

**Fig. 2:**
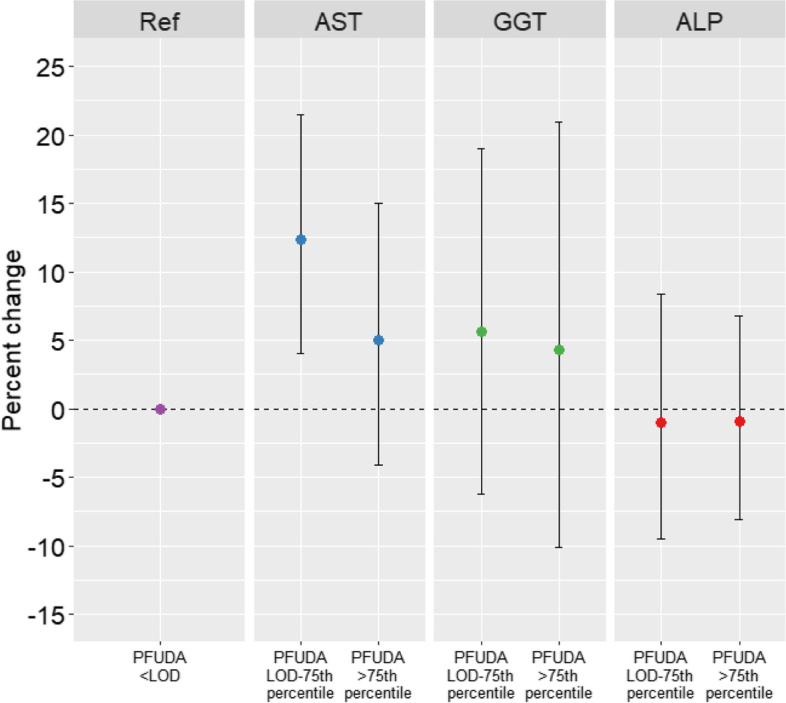
Percent change (95%CI) in AST, GGT, and ALP between categorized levels of PFUDA. The reference group consists of those participants with concentrations below the limit of detection (LOD)

**Fig. 3:**
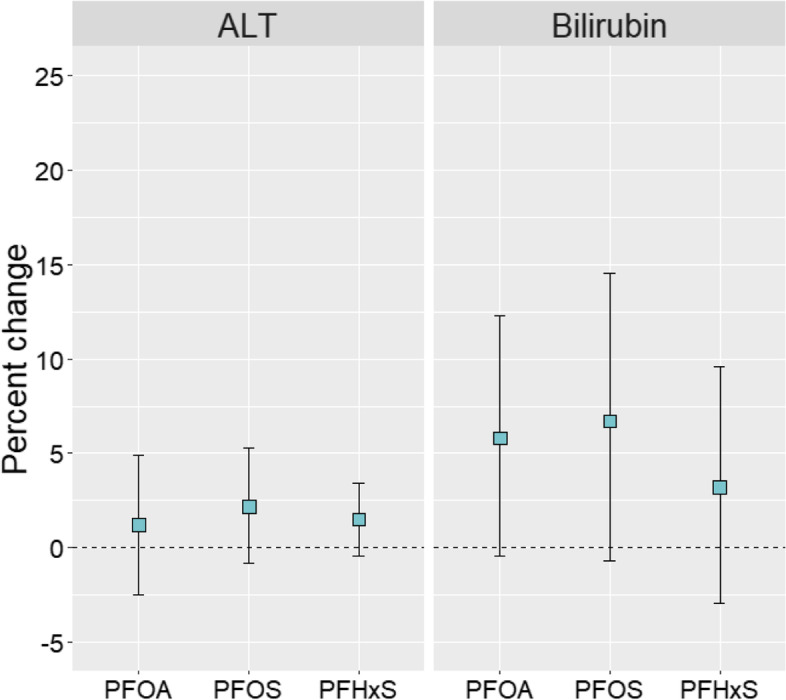
Percent change (95%CI) in ALT and total bilirubin for PFOA, PFOS, and PFHxS. Coefficients for individual PFAS represent the percent change for each two-fold increase in PFAS concentrations

The association between PFOA and GGT was considerably stronger, and statistically significant, among individuals obtaining less than 150 vs. 150 min or more of moderate-to-vigorous physical activity per week (16.5% vs. 6.6%). Each two-fold increase in plasma PFOS concentrations was associated with 7.6% (95%CI: 3.0, 12.4) higher GGT and 4.1% (95%CI: 2.4, 5.9) higher ALP. The association between PFOS and AST was stronger among males vs. females (7.6% vs. 3.3%). Each two-fold increase in plasma PFHxS concentrations was associated with 3.1% (95%CI: 1.9, 4.4) higher AST and 3.9% (95%CI: 1.2, 6.6) higher GGT. The association between PFHxS and ALP was only apparent among normal/under weight individuals, which was significantly different from those with obesity (5.9% vs. 0.2%), but not significantly different from overweight individuals. Each two-fold increase in plasma PFNA concentrations was associated with 6.4% (95%CI: 3.8, 9.0) higher AST, 13.8% (95%CI: 4.2, 24.3) higher GGT, and 3.2% (95%CI: 0.3, 6.3) higher ALP. Associations for PFNA did not differ by sex, BMI, or physical activity levels. No associations were observed for PFDA. Participants with categorized PFUDA concentrations between the LOD and 75^th^ percentile had significantly higher level of AST concentrations compared to those participants with concentrations less than the LOD (Fig. 2). No difference was observed for participants with PFUDA concentrations > 75^th^ percentile. No associations were observed with ALT or total bilirubin (Fig. 3).

In quantile g-computation models, each simultaneous one-quartile increase in the mixture of PFOA, PFOS, PFHxS, PFNA, and PFDA was associated with 7.5% (95%CI: 4.0, 10.4) higher AST, 9.7% (95%CI: 1.7, 17.0) higher GGT, and 2.8% (95%CI: 0.5, 5.4) higher ALP (Fig. 1 and Additional File 1, see Supplemental Table 6)). Generally, PFOA and PFNA provided the strongest positive weights (up to 0.53 for PFOA and 0.50 for PFNA) while PFDA consistently provided the strongest negative weight (Additional File 1, see Supplemental Table 6).

Results were similar after excluding 150 individuals with reported liver disease in a sensitivity analysis (Additional File 1, see Supplemental Table 7), except for the association between PFOS and total bilirubin which was statistically significant, although with a similar effect size compared to the main analysis (7.3% change; 95%CI: 0.4, 14.7).

## Discussion

In this paper, we examined associations between six PFAS and five liver function biomarkers in a representative sample of Canadian adults from three cycles of the CHMS. We showed that plasma concentrations of PFOA, PFOS, PFHxS, and PFNA were consistently positively associated with serum concentrations of AST, GGT, and ALP. These results are generally consistent with previous epidemiological studies demonstrating deleterious associations between PFAS and liver function biomarkers in both highly exposed [22–24] and general populations [25–29]. However, this literature has focused on individual effects whereas people are routinely exposed to a mixture of PFAS [12]. We extend this literature by demonstrating that the combination, or mixture, of these PFAS is also associated with liver function biomarkers. In quantile g-computation, the weights represent the independent, adjusted beta coefficients (scaled to between -1 and + 1) for the individual quantized exposures. The weights will suffer from the same variance inflation issues as other methods which estimate independent effects (e.g., multiple regression). Weights can be thoughts of as representing the proportion of the negative or positive partial effect due to a specific exposure. Generally, PFOA and PFNA contributed the strongest positive weights in the quantile g-computation models while PFDA consistently contributed the strongest negative weight. This is in line with associations observed in the individual PFAS models where associations for PFOA and PFNA were generally the strongest among the six PFAS, and associations for PFDA were generally the weakest.

The liver function biomarkers examined in this paper are useful clinical indicators of overall liver injury and are commonly used to diagnose liver diseases, including non-alcoholic fatty liver disease [15, 16]. We observed consistent associations with GGT and ALP, which are both early markers of cholestatic liver disorders including biliary obstruction and cholestasis [50]. For total bilirubin, another indicator of such conditions, we observed similar effect sizes but only the association with PFOS was statistically significant after excluding participants with existing reported liver disease. This is consistent with evidence from a cohort study examining liver biopsies which demonstrated that PFAS can impact metabolic pathways involved in bile acid metabolism [51]. We also observed consistent positive associations with AST, a non-specific marker of hepatocyte damage that is released into the circulation following hepatocyte death [52]. PFAS are associated with biomarkers of hepatocyte apoptosis [53], which supports this finding. However, we did not observe associations for ALT, a specific marker of hepatocyte damage, which is not consistent with previous analyses in the US or China [22–25, 27, 28]. In these studies, the concentrations of PFOA and PFOS were higher than in the CHMS, which may explain this discrepancy.

A previous analysis of CHMS data by Cakmak et al. (2022) similarly demonstrated associations between several PFAS and GGT, but only PFOA was associated with AST. Contrary to our results, they identified some positive associations with ALT and total bilirubin and also identified negative associations between PFOS and ALP as well as between PFHxS and GGT (among those aged > 33 years old in an age-stratified analysis). They did not identify interactions with sex or BMI. These discrepancies could be because we restricted the analytical sample to adult participants (vs. participants aged 3–79 as in Cakmak et al. (2022)), we additionally adjusted for accelerometer-measured physical activity levels, we had a lower sample size in our paper as a result of restricting our analysis for the subset of individuals who participated in the accelerometer portion of the CHMS, and we did not include recruitment site as a random effect in our models. Despite these differences, both of these analyses provide much needed Canadian evidence and our results are generally consistent with the broader literature supporting the role of PFAS as a hepatotoxin [11, 12].

The strongest association that we observed was among physically inactive adults – each two-fold increase in PFOA concentrations was associated with 16.5% higher GGT concentrations. This association was twice as strong – or 9.9% higher, in absolute terms – compared to those adults meeting Canada’s physical activity guidelines. This suggests that higher levels of physical activity may be protective against the hepatotoxic effects of PFOA. This is consistent with results from a randomized clinical trial of a two-year lifestyle intervention on weight change, which included maintaining > 150 min of moderate-to-vigorous physical activity. These authors showed that the deleterious effects of PFAS, and PFOA in particular, on cholesterol and triglycerides was apparent only in the physically inactive control group, and not in the physically active intervention group [33]. Similar findings have been observed from this intervention study for obesity [31], and incident diabetes [32]. One explanation for our finding is that higher levels of exposure to PFOA may modify individuals’ sensitivity to other risk factors for liver diseases. This is consistent with the “two-hit” hypothesis, whereby exposure to an environmental chemical may compromise the liver’s protective responses against other lifestyle risk factors [13]. This has primarily been studied in the context of chemical-nutrition interaction effects (e.g., hypercaloric or high-fats diets) [12], but physical inactivity could play a similar role. Ultimately, further research is needed to corroborate this finding and to investigate the role of physical activity as a potential modifier of other associations between PFAS and health. Accelerometers are considered the gold-standard field-based measure of physical activity [54]. The use of objectively measured physical activity data from accelerometers is a strength of the current study, and should be considered for use in future analyses.

We found that the association between PFOA and AST was twice as strong among men vs. women. The hepatotoxic effects of PFAS may be sex-specific because of sexually dimorphic responses to PFAS exposure [11], lipid metabolism, and expression of several nuclear receptors involved in energy homeostasis [55]. In addition, this could be because menstruation [55, 56], pregnancy (via transplacental transfer [56]), and breastfeeding [57] are all prominent excretion pathways for PFAS for women which would reduce their overall PFAS body burden [58, 59]. However, previous epidemiological evidence for sex-specific associations between PFAS and liver function biomarkers is equivocal [12]. Using data from adolescents in the US National Health and Nutrition Examination Survey (NHANES), Attanasio (2019) reported deleterious, though inconsistent, associations between PFAS and liver function biomarkers among female adolescents, and largely mixed associations among male adolescents. However, sex-specific associations were not observed in a previous analysis of participants aged 3–79 in the CHMS [29], as well as an analysis of adult participants exposed to high levels of PFOA in the C8 Health Study [23].

We found that the association between PFHxS and ALP was stronger among individuals with normal/underweight vs. those with obesity. Obesity can induce lipid accumulation in the liver and is a risk factor for non-alcoholic fatty liver disease [60]. Therefore, we would have expected to see stronger associations among individuals with obesity. However, obesity has been be associated with higher ALP concentrations [61, 62], so it is also possible that obesity could obscure the relationship with PFHxS. Two previous analyses of NHANES data identified associations between PFOA [25] as well as PFHxS and PFNA [27] and liver function biomarkers among individuals with obesity. However, Lin et al. (2010) did not examine ALP, Jain and Ducatman (2019) did not observe associations with ALP, and neither study formally tested for interaction across BMI categories. A previous analysis of CHMS data did not identify effect modification by BMI for these liver function biomarkers [29]. None of these studies adjusted for objectively measured physical activity levels, which could explain the discrepancy with our findings.

This study has several limitations. The cross-sectional design of the CHMS precludes us from establishing temporality, a critical component of causality. However, given the long half-lives of these PFAS [63], plasma concentrations may reflect long term exposure. In addition, we cannot rule out the possibility of reverse causality if PFAS tend to bioaccumulate more (either through altered distribution or elimination) in individuals with existing markers of liver damage. We attempted to minimize this concern in a sensitivity analysis by excluding individuals with reported liver disease, but this does not eliminate the possibility that higher PFAS concentrations could be the result of more advanced, yet still sub-clinical, liver disease. However, when interpreting our findings within the context of longitudinal and experimental evidence demonstrating the hepatotoxic effects of PFAS, as summarized in a recent systematic review and meta-analysis [12], it is unlikely that our findings are the result of reverse causality. Not all of the PFAS and liver function biomarkers were measured across all cycles of the CHMS. As a result, in the mixture model we opted to include PFNA and PFDA and to examine associations with a smaller number of outcomes. This meant we did not examine associations with a smaller mixture on ALT and total bilirubin, for which the individual results were null in the main analysis. Finally, total bilirubin represents the sum of both direct (conjugated) and indirect (unconjugated) bilirubin, which were not measured individually in the CHMS. This is a limitation, since direct bilirubin is likely a more relevant marker of effect for PFAS-related hepatotoxicity. This may help to explain the relatively wide confidence intervals that we observed for the null associations with total bilirubin in this analysis. Future studies should consider using direct, rather than total, bilirubin.

## Conclusion

In this nationally representative sample of Canadian adults, we showed that higher plasma concentrations of PFOA, PFOS, PFHxS, and PFNA – both individually and as a mixture – were associated with higher serum concentrations of the liver function biomarkers AST, GGT, and ALP. We show, for the first time, that higher levels of physical activity appear to be protective against the hepatotoxic effects of PFOA. Future research is warranted to investigate the stability of this finding in other populations. This work contributes to a growing body of evidence supporting the hepatotoxic effects of PFAS.

## Supplementary Information

**Additional file 1:** **Supplemental Table 1.** Limits of detection (µg/L) for plasma concentrations of six perfluoroalkyl substances. **Supplemental Table 2.** Sample sizes for analyses of PFAS and liver function biomarkers across cycles 1 (2007-2009), 2 (2009-2011), and 5 (2016-2017) of the Canadian Health Measures survey. **Supplemental Table 3.** Descriptive statistics for perfluoroalkyl substances and sociodemographic characteristics stratified by sex. **Supplemental Table 4.** Pearson correlations between Log_2_-transformed plasma concentrations of perfluoroalkyl substances. **Supplemental Table 5.** Percent difference (95%CI) in liver function biomarkers for individual PFAS. **Supplemental Table 6.** Percent difference (95%CI) in liver function biomarkers for a mixture of PFAS. **Supplement Table 7.** Sensitivity analysis of percent difference (95%CI) in liver function biomarkers for individual PFAS. **Supplement Table 8.** Description of liver diseases reported by 150 individuals in cycles 1, 2, and 5 of the Canadian Health Measures Survey.

## Data Availability

The data from the Canadian Health Measures Survey (CHMS) are publicly available here: https://www.statcan.gc.ca/eng/statistical-programs/document/5071_D5_T9_V1.

## Abbreviations

ALP: Alkaline phosphatase
ALT: Alanine aminotransferase
AST: Aspartate aminotransferase
BMI: Body mass index
CHMS: Canadian Health Measures Survey
GGT: Gamma-glutamyltransferase
LOD: Limit of detection
PFDA: Perfluorodecanoic acid
PFHxS: Perfluorohexane sulfonate
PFNA: Perfluorononanoic acid
PFOA: Perfluoroctanoic acid
PFOS: Perfluorooctane sulfonate
PFUDA: perfluoroundecanoic acid
