# Supplementary material for: Individual and mixture associations of perfluoroalkyl substances on liver function biomarkers in the Canadian Health Measures Survey
Source: Environ Health. 2022 Sep 14;21:85. doi: 10.1186/s12940-022-00892-6 (PMC9472375; doi:10.1186/s12940-022-00892-6)
Supplement: Supplementary file 1 — Additional file 1: Supplemental Table 1. Limits of detection (µg/L) for plasma concentrations of six perfluoroalkyl substances. Supplemental Table 2. Sample sizes for analyses of PFAS and liver function biomarkers across cycles 1 (2007-2009), 2 (2009-2011), and 5 (2016-2017) of the Canadian Health Measures survey. Supplemental Table 3. Descriptive statistics for perfluoroalkyl substances and sociodemographic characteristics stratified by sex. Supplemental Table 4. Pearson correlations between Log2-transformed plasma concentrations of perfluoroalkyl substances. Supplemental Table 5. Percent difference (95%CI) in liver function biomarkers for individual PFAS. Supplemental Table 6. Percent difference (95%CI) in liver function biomarkers for a mixture of PFAS. Supplement Table 7. Sensitivity analysis of percent difference (95%CI) in liver function biomarkers for individual PFAS. Supplement Table 8. Description of liver diseases reported by 150 individuals in cycles 1, 2, and 5 of the Canadian Health Measures Survey. [file 12940_2022_892_MOESM1_ESM.docx]

Supplemental Table 1 – Limits of detection (µg/L) for plasma concentrations of six perfluoroalkyl substances

|  | Cycle 1  (2007-2009) | Cycle 2  (2009-2011) | Cycle 5  (2016-2017) |
| --- | --- | --- | --- |
| Perfluorooctanoic acid (PFOA) | 0.30 | 0.10 | 0.066 |
| Perfluorooctane sulfonate (PFOS) | 0.30 | 0.30 | 0.430 |
| Perfluorohexane sulfonate (PFHxS) | 0.30 | 0.20 | 0.063 |
| Perfluorononanoic acid (PFNA) | - | 0.20 | 0.130 |
| Perfluorodecanoic acid (PFDA) | - | 0.10 | 0.092 |
| Perfluoroundecanoic acid (PFUDA) | - | 0.09 | 0.120 |

*Data from cycles 1, 2, and 5 of the Canadian Health Measures Survey.*

Supplemental Table 2 – Sample sizes for analyses of PFAS and liver function biomarkers across cycles 1 (2007-2009), 2 (2009-2011), and 5 (2016-2017) of the Canadian Health Measures survey.

|  | AST | GGT | ALP | ALT | Total bilirubin |
| --- | --- | --- | --- | --- | --- |
| PFOA | 3572 | 3574 | 3556 | 2682 | 2854 |
| PFOS | 3573 | 3575 | 3557 | 2682 | 2854 |
| PFHxS | 3572 | 3574 | 3556 | 2681 | 2853 |
| PFNA | 1403 | 1404 | 1403 | - | - |
| PFDA | 1385 | 1386 | 1385 | - | - |
| PFUDA | 1431 | 1432 | 1431 | - | - |

Sample sizes differ as a result of differences in data availability across cycles 1 (2007-2009), 2 (2009-2011), and 5 (2016-2017) of the Canadian Health Measures survey

Supplemental Table 3 – Descriptive statistics for perfluoroalkyl substances and sociodemographic characteristics stratified by sex

|  | Males (n = 2290) | | Females (n = 2367) | |
| --- | --- | --- | --- | --- |
|  | n | %^a^ | n | %^a^ |
| Age | | | | |
| 20-29 years | 304 | 21 | 291 | 13 |
| 30-39 years | 484 | 20 | 583 | 24 |
| 40-49 years | 529 | 21 | 507 | 20 |
| 50-59 years | 299 | 18 | 327 | 21 |
| 60-74 years | 674 | 19 | 659 | 22 |
| Household income category | | | | |
| Lowest income | 565 | 25 | 758 | 25 |
| Lower middle | 418 | 16 | 477 | 21 |
| Upper middle | 622 | 27 | 518 | 24 |
| Highest income | 625 | 32 | 548 | 30 |
| Race | | | | |
| White | 1867 | 80 | 1969 | 79 |
| Not white | 364 | 20^c^ | 333 | 21 |
| Level of education completed | | | | |
| Less than Secondary | 269 | 11 | 246 | 8.8 |
| Secondary | 392 | 22 | 401 | 19 |
| At least some Post-Secondary | 1599 | 67 | 1700 | 72 |
| Body mass index category | | | | |
| Under/normal Weight | 663 | 35 | 1023 | 46 |
| Overweight | 1018 | 39 | 695 | 29 |
| Obese | 603 | 26 | 644 | 25 |
| Smoking status | | | | |
| Current | 513 | 27 | 441 | 17 |
| Former | 729 | 31 | 696 | 27 |
| Never | 1042 | 42 | 1226 | 56 |
| Alcohol consumption | | | | |
| Regular consumption | 1734 | 78 | 1491 | 63 |
| Occasional consumption | 254 | 11 | 468 | 20 |
| Former consumption | 222 | 7.2 | 254 | 8.8 |
| Never consumed | 80 | 3.1^b^ | 153 | 8.3^b^ |
| Dyslipidemia | | | | |
| Yes | 603 | 36 | 452 | 25 |
| No | 981 | 64 | 1218 | 75 |
| Type II diabetes | | | | |
| Yes | 128 | 5 | 102 | 4 |
| No | 2160 | 95 | 2259 | 96 |
|  | n | Mean (SE) | n | Mean(SE) |
| Average reported daily moderate-to-vigorous physical activity (minutes/week) | 1848 | 190 (11) | 1978 | 140 (5.6) |

*Data from cycles 1, 2, and 5 of the Canadian Health Measures Survey.*

*SE – standard error*

*^a^ percent within column calculated using sample weights*

*^b^ Interpret with caution. Coefficient of variation is between 16.6% and 33.3%*

Supplemental table 4 – Pearson correlations between Log_2_-transformed plasma concentrations of perfluoroalkyl substances

|  | PFOS | PFHxS | PFNA | PFDA |
| --- | --- | --- | --- | --- |
| PFOA | 0.70 | 0.63 | 0.68 | 0.40 |
| PFOS |  | 0.68 | 0.71 | 0.54 |
| PFHxS |  |  | 0.42 | 0.20 |
| PFNA |  |  |  | 0.68 |

*Data from cycles 1, 2, and 5 of the Canadian Health Measures Survey.*

Supplemental Table 5 –Percent difference (95%CI) in liver function biomarkers for individual PFAS

|  | AST | GGT | ALP | ALT | Total bilirubin |
| --- | --- | --- | --- | --- | --- |
| PFOA (µg/L) | 7.9 (5.4, 10.4) | - | 3.9 (1.7, 6.1) | 1.2 (-2.5, 4.9) | 5.8 (-0.4, 12.3) |
| Physically active ^a^ | - | 6.6 (-1.6, 15.5) | - | - | - |
| Not physically active | - | 16.5 (10.4, 23.0)* | - | - | - |
| PFOS (µg/L) | - | 7.6 (3.0, 12.4) | 4.1 (2.4, 5.9) | 2.2 (-0.8, 5.3) | 6.7 (-0.7, 14.5) |
| Male | 7.6 (4.6, 10.8)† | - | - | - | - |
| Female | 3.3 (1.2, 5.5) | - | - | - | - |
| PFxHS (µg/L) | 3.1 (1.9, 4.4) | 3.9 (1.2, 6.6) | - | 1.5 (-0.4, 3.4) | 3.2 (-2.9, 9.6) |
| Normal/under weight | - | - | 5.9 (2.8, 9.1)‡ | - | - |
| Overweight | - | - | 2.5 (-0.4, 5.6) | - | - |
| Obese | - | - | 0.2 (-3.4, 3.9) | - | - |
| PFNA (µg/L) | 6.4 (3.8, 9.0) | 13.8 (4.2, 24.3) | 3.2 (0.3, 6.3) | - | - |
| PFDA (µg/L) | 1.7 (-1.1, 4.4) | 3.3 (-4.8, 11.9) | -0.8 (-3.1, 1.5) | - | - |
| PFUDA (µg/L) | | | | | |
| <LOD | reference | reference | reference | - | - |
| LOD – 75^th^ percentile | 12.4 (4.0, 21.5) | 5.6 (-6.2, 19.0) | -1.0 (-9.5, 8.4) | - | - |
| >75^th^ percentile | 5.0 (-4.1, 15.0) | 4.3 (-10.1, 20.9) | -0.9 (-8.1, 6.8) | - | - |

Coefficients for PFOA, PFOS, PFHxS, PFNA, and PFDA represent the percent change for each 2-fold increase. Coefficients for PFUDA represent the difference in percent change from the reference group (categorical).

All models are adjusted for age, sex, race, BMI, alcohol consumption, smoking, level of education, household income, and average minutes/day of moderate-to-vigorous physical activity

^a^ Defined as meeting the Canadian physical activity guidelines which recommend >150 min/week of moderate-to-vigorous physical activity

* – significantly different from those meeting physical activity guidelines

† – significantly different from female

‡ – significantly different from obese

Supplemental Table 6 – Percent difference (95%CI) in liver function biomarkers for a mixture of PFAS

|  | Percent difference (95% CI) | Weights | | | | |
| --- | --- | --- | --- | --- | --- | --- |
|  |  | PFOA | PFOS | PFHxS | PFNA | PFDA |
| AST | 7.5 (4.0, 10.4) | 0.53 | 0.16 | 0.12 | 0.18 | -1.0 |
| GGT | 9.7 (1.7, 17.0) | 0.42 | 0.20 | -0.21 | 0.38 | -0.79 |
| ALP | 2.8 (0.5, 5.4) | -0.12 | 0.34 | 0.16 | 0.50 | -0.88 |

Coefficients represent a simultaneous one-quartile increase in the mixture of PFOA, PFOS, PFHxS, PFNA, and PFDA using quantile g-computation. All models are adjusted for age, sex, race, BMI, alcohol consumption, smoking, level of education, household income, and average minutes/day of moderate-to-vigorous physical activity.

Supplement table 7 – Sensitivity analysis of percent difference (95%CI) in liver function biomarkers for individual PFAS

|  | AST | GGT | ALP | ALT | Total bilirubin |
| --- | --- | --- | --- | --- | --- |
| PFOA (µg/L) | 7.9 (5.4, 10.4) | - | 3.9 (1.7, 6.1) | 1.2 (-2.4, 5.0) | 3.8 (-2.1, 9.9) |
| Physically active ^a^ | - | 7.3 (-1.1, 16.4) | - | - | - |
| Not physically active | - | 16.5 (10.3, 23.0)* | - | - | - |
| PFOS (µg/L) | - | 7.7 (3.1, 12.6) | 4.2 (2.4, 5.9) | 1.8 (-1.6, 5.4) | 7.3 (0.4, 14.7) |
| Male | 7.7 (4.9, 10.5) | - | - | - | - |
| Female | 3.3 (1.0, 5.6) † | - | - | - | - |
| PFHS (µg/L) | 3.2 (1.8, 4.5) | 4.0 (1.3, 6.8) | - | 1.5 (-0.4, 3.5) | 3.3 (-3.1, 10.0) |
| Normal/under weight | - | - | 5.8 (2.7, 9.0) ‡ | - | - |
| Overweight | - | - | 2.7 (-0.5, 6.0) | - | - |
| Obese | - | - | 0.01 (-3.6, 3.8) | - | - |
| PFNA (µg/L) | 6.6 (3.9, 9.3) | 14.3 (4.5, 25.0) | 3.4 (0.4, 6.4) | - | - |
| PFDA (µg/L) | 1.9 (-0.9, 4.8) | 3.3 (-4.8, 12.1) | -0.8 (-3.1, 1.5) | - | - |
| PFUDA (µg/L) | | | | | |
| <LOD | reference | reference | reference | - | - |
| LOD – 75^th^ percentile | 11.9 (3.9, 20.6) | 6.1 (-5.8, 19.5) | -1.0 (-9.8, 8.6) | - | - |
| >75^th^ percentile | 5.0 (-4.3, 15.2) | 3.7 (-10.7, 20.4) | -0.9 (-8.1, 6.8) | - | - |

Excludes 150 individuals with liver disease

Coefficients for PFOA, PFOS, PFHxS, PFNA, and PFDA represent the percent change for each 2-fold increase. Coefficients for PFUDA represent the difference in percent change from the reference group (categorical).

All models are adjusted for age, sex, race, BMI, alcohol consumption, smoking, level of education, household income, and average minutes/day of moderate-to-vigorous physical activity

^a^ Defined as meeting the Canadian physical activity guidelines which recommend >150 min/week of moderate-to-vigorous physical activity

* – significantly different from those meeting physical activity guidelines

† – significantly different from female

‡ – significantly different from obese

Supplement table 8 – Description of liver diseases reported by 150 individuals in cycles 1, 2, and 5 of the Canadian Health Measures Survey

|  | n | % |
| --- | --- | --- |
| Hepatitis A | | |
| Yes | 1 | 0.7 |
| No | 144 | 99.3 |
| Hepatitis B | | |
| Yes | 5 | 3.5 |
| No | 140 | 96.5 |
| Hepatitis C | | |
| Yes | 11 | 7.6 |
| No | 134 | 92.4 |
| Jaundice | | |
| Yes | 5 | 3.5 |
| No | 140 | 96.5 |
| Cirrhosis | | |
| Yes | 6 | 4.1 |
| No | 139 | 95.9 |
| Gallstones | | |
| Yes | 74 | 51.0 |
| No | 71 | 49.0 |
| Other | | |
| Yes | 52 | 35.9 |
| No | 93 | 64.1 |

Individuals may have reported having more than one condition
